# Performance of a RT-PCR Assay in Comparison to FISH and Immunohistochemistry for the Detection of ALK in Non-Small Cell Lung Cancer

**DOI:** 10.3390/cancers9080099

**Published:** 2017-08-01

**Authors:** David R. Hout, Brock L. Schweitzer, Kasey Lawrence, Stephan W. Morris, Tracy Tucker, Rosetta Mazzola, Rachel Skelton, Frank McMahon, John Handshoe, Mary Lesperance, Aly Karsan, David L. Saltman

**Affiliations:** 1 Insight Genetics, Inc., Suite 510, 2 International Plaza, Nashville, TN 37217, USA; dhout@insightgenetics.com (D.R.H.); bschweitzer@insightgenetics.com (B.L.S.); klawrence@insightgenetics.com (K.L.); smorris@insightgenetics.com (S.W.M.); rachelleahskelton@gmail.com (R.S.); fmcmahon@insightgenetics.com (F.M.); jhandshoe@insightgenetics.com (J.H.); 2 Department of Pathology and Laboratory Medicine, BC Cancer Agency, 675 West 10th Avenue, Vancouver, BC V5Z 1L3, Canada; ttucker2@bccancer.bc.ca (T.T.); akarsan@bccancer.bc.ca (A.K.); 3 Department of Medical Oncology, British Columbia Cancer Agency, VIC 2410 Lee Avenue, Victoria, BC V8R 6V5, Canada; rosetta.mazzola@alumni.ubc.ca; 4 Department of Mathematics and Statistics, University of Victoria, Box 1700, STN CSC, Victoria, BC V8W 2Y2, Canada; mlespera@uvic.ca

**Keywords:** anaplastic lymphoma kinase, fluorescence in situ hybridization, immunohistochemistry, non-small cell lung cancer, reverse transcriptase-polymerase chain reaction

## Abstract

Patients with lung cancers harboring an activating anaplastic lymphoma kinase (*ALK*) rearrangement respond favorably to ALK inhibitor therapy. Fluorescence in situ hybridization (FISH) and immunohistochemistry (IHC) are validated and widely used screening tests for *ALK* rearrangements but both methods have limitations. The ALK RGQ RT-PCR Kit (RT-PCR) is a single tube quantitative real-time PCR assay for high throughput and automated interpretation of *ALK* expression. In this study, we performed a direct comparison of formalin-fixed paraffin-embedded (FFPE) lung cancer specimens using all three ALK detection methods. The RT-PCR test (diagnostic cut-off Δ*C*_t_ of ≤8) was shown to be highly sensitive (100%) when compared to FISH and IHC. Sequencing of RNA detected full-length *ALK* transcripts or *EML4-ALK* and *KIF5B-ALK* fusion variants in discordant cases in which *ALK* expression was detected by the ALK RT-PCR test but negative by FISH and IHC. The overall specificity of the RT-PCR test for the detection of ALK in cases without full-length *ALK* expression was 94% in comparison to FISH and sequencing. These data support the ALK RT-PCR test as a highly efficient and reliable diagnostic screening approach to identify patients with non-small cell lung cancer whose tumors are driven by oncogenic ALK.

## 1. Introduction

The anaplastic lymphoma kinase (*ALK*) gene was discovered in 1994 as the fusion partner of the nucleophosmin (*NPM1*) gene in the recurrent t(2;5) chromosomal translocation found in anaplastic large-cell lymphoma (ALCL) [1]. Since the original description of the *NPM1-ALK* fusion gene, a large number of distinct *ALK* fusion partners have been identified in ALCL, as well as a variety of other malignancies. The discovery of the *EML4-ALK* fusion gene in 2007 [2] as a driver of the malignant phenotype in a small subset of non-small cell lung cancers (NSCLC) led to the accelerated development of the first ALK tyrosine kinase inhibitor, crizotinib, which was given regulatory approval by the FDA in 2011 together with the Vysis ALK Break Apart Fluorescence in situ hybridization (FISH) Probe Kit (Abbott Molecular, Des Plaines, IL, USA) companion diagnostic assay [3]. Treatment of NSCLC patients with ALK inhibitors is associated with high response rates and prolonged progression-free survival in comparison to cytotoxic chemotherapy [4,5].

Although FISH is still commonly used as a screening test for *ALK* rearrangements or for confirmation of equivocal immunohistochemistry (IHC) results, it has several disadvantages [6,7]. These include the high cost associated with the technique, the need for specific expertise for interpreting the results, and the long turn-around time. In addition, there are a number of technical issues that make FISH challenging as an accurate *ALK* fusion detection assay and contribute to false-positive and -negative results. Most fusions involve an intrachromosomal inversion of *EML4* and *ALK*, two genes that are in close proximity on chromosome 2p. Distinguishing split signals from the normal fused pattern seen with wild-type *ALK* can be challenging, as can be the interpretation of atypical signal patterns. Further, the cutoff of 15% or greater positive cells has been widely tested but is another confounding factor that can contribute to false-negative samples using FISH, particularly for specimens with a low tumor content. Despite these challenges, FISH is still regarded as the gold standard assay for the detection of *ALK* rearrangements and a comparator for the validation of other ALK detection methods.

IHC is widely used as an initial screening test for ALK involvement in NSCLC in Canada, Europe, and more recently, in the United States. One of the main advantages of IHC in comparison to FISH and RT-PCR is the detection of the ALK protein, which is the target of ALK inhibitors. Although some studies suggest IHC is more sensitive than FISH for detecting ALK fusions [8], FISH-positive/IHC-negative cases with responses to ALK inhibitors have been reported in the literature [9]. Other advantages of IHC are its low cost, short turn-around time, and ease of use. IHC-positive samples may require confirmation with FISH because of the low positive predictive value associated with lower IHC staining intensities. In addition to problems that may arise in the scoring systems of ALK IHC, discordance between laboratories can occur due to a lack of standardization of reagents and training [10]. The Ventana ALK (D5F3) CDx Assay (Ventana Medical Systems, Tucson, AZ, USA) was approved by the FDA in 2015 as a companion detection test for the use of crizotinib [11]. Another commonly used and validated antibody for the detection of *ALK* fusions in NSCLC is the Novocastra monoclonal antibody 5A4 (Leica, Wetzlar, Germany) [12,13].

**Figure 1:**
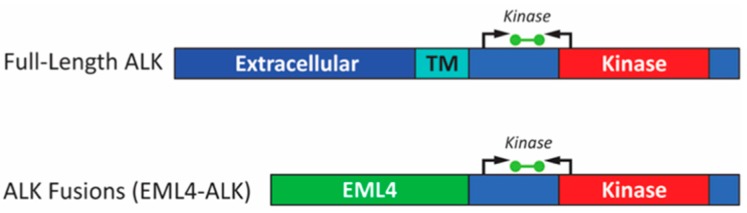
Schematic of full-length *ALK* and *EML4-ALK* fusion transcripts indicating location of ALK RT-PCR assay. Regions of the transcript which encode domains in the ALK protein are indicated: Extracellular domain of receptor (Extracellular), transmembrane domain (TM), tyrosine kinase domain (Kinase), echinoderm microtubule associated protein like 4 (*EML4*) fusion gene partner. Arrows indicate location of RT-PCR primers and green bar indicates fluorescent probe.

RT-PCR-based assays have not been as widely used as FISH and IHC for the detection of *ALK* rearrangements in NSCLC. However, recent studies using FISH and IHC or FISH alone in comparison to RT-PCR have demonstrated that RT-PCR has a high sensitivity and specificity [14,15,16]. These tests have the advantages of a rapid turn-around time and ease of analysis, and can be used on biopsy or cytology specimens with lower tumor content than needed for accurate FISH and IHC testing [14,15,17]. In the study described in this report, we compare a commercially available RT-PCR assay (ALK RGQ RT-PCR Kit, QIAGEN, Manchester, UK) designed to identify mRNA produced by all *ALK* rearrangements regardless of the fusion partner or variant (Figure 1), with IHC that detects the ALK protein and FISH that directly identifies *ALK* genomic rearrangements in FFPE samples from patients with NSCLC.

## 2. Results

### 2.1. Clinicopathological Characteristics

A total of 95 cases had sufficient tumor content and quality to be assessed by IHC, FISH and RT-PCR (Table 1). The median age was similar in the RT-PCR-positive and -negative groups, 69 and 69.5 years, respectively. The majority (76%) of tumor samples were biopsies and cytology, while twenty-four percent were resections including archival lung, brain metastases, and one case of nephrectomy for a renal mass that was proven to be metastatic NSCLC. Fifty-five (58%) samples were obtained from primary lung tumor, while 40 (42%) were from metastatic lesions including mediastinal lymph nodes. There was no significant difference between the percentage of samples from lung primary tumors versus samples from metastatic sites in the RT-PCR-positive group compared to the RT-PCR, IHC and FISH-positive group. All sample histologies were adenocarcinomas with the exception of two cases of adenosquamous tumors from the ALK RT-PCR-positive group. All patients had either stage IIIB or IV disease and all tumors were EGFR wild-type. There was a significantly higher proportion of never or light smokers (50%) in the ALK RT-PCR-positive cohort compared to the RT-PCR-negative cases (1.7%). The group of patients whose tumors were ALK-positive by RT-PCR, IHC and FISH contained 76% never or light smokers.

### 2.2. Sensitivity and Specificity of RT-PCR Compared to FISH

Thirty-six samples were found to be positive by RT-PCR using a Δ*C*_t_ cut-off of ≤8 (Table 2). Of these 36 samples, 22 cases were IHC-positive (including 19 samples that were IHC 2+ or 3+). One sample was not tested by IHC but was FISH-positive. Twenty samples were FISH-positive. One sample was 3+ IHC but failed testing by FISH twice.

This sample was assumed to be FISH-positive based on the experience from previously reported studies in which 100% of IHC 3+ have been FISH-positive [18]. Using FISH as a reference, the sensitivity of RT-PCR was 100% (95% CI: 70–99%), while the specificity was 80% (95% CI: 69–88%) (Table 3). Next generation sequencing (NGS) and or Sanger sequencing was performed on all samples from which there was sufficient quality and quantity of specimen. Eleven of the 15 discordant samples (73%) that were RT-PCR-positive but FISH-negative were found to have *ALK* rearrangements, including eight *EML4-ALK* and three *KIF5B-ALK* variants. The other four discordant samples expressed full-length (wild-type) *ALK*. Using either FISH or detection of an *ALK* rearrangement by sequencing as a positive reference, the sensitivity of RT-PCR with a Δ*C*_t_ ≤8 cut-off value was 100% (95% CI: 89–100%) and the specificity 94% (95% CI: 85–98%) when these 4 full-length *ALK*-expressing cases were excluded (Table 4).

If a lower Δ*C*_t_ of ≤3.5 was used as the cut-off value with FISH for a reference, as previously reported by Marchetti et al. [16], the specificity increased to 99% (95% CI: 93–100%) but the sensitivity decreased to 90% (95% CI: 70–99%) (Table 4). The sensitivity of RT-PCR dropped to 62% when using a Δ*C*_t_ of ≤3.5 and FISH or sequencing as a positive reference largely due to ten cases with Δ*C*_t_ values > 3.5 but < 8 that demonstrated fusions on sequencing (Table 2). There were also two cases that were RT-PCR and FISH-positive with Δ*C*_t_ values > 3.5 but < 8. Case 15 (Table 2) had a Δ*C*_t_ of 7.12 but was FISH-positive with an IHC of 1+. A second sample (case 20) had a Δ*C*_t_ of 3.62 but was FISH-positive with an IHC 3+.

### 2.3. Correlation of RT-PCR, FISH and IHC with Response to ALK Inhibitor Therapy

**Figure 2:**
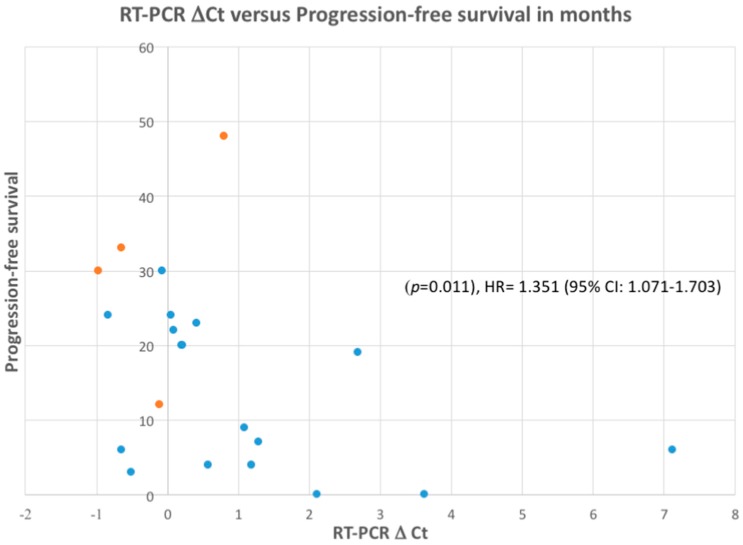
Association between RT-PCR Δ*C*_t_ and progression-free survival (months) after starting the first-line ALK inhibitor crizotinib for ALK fusion-positive non-small cell lung cancer. Red dots represent cases that continue to respond to crizotinib at the time of data analysis.

All patients who were FISH-positive or who failed FISH but were 3+/3 IHC were eligible for treatment with the first-generation ALK inhibitor crizotinib. The response data including progression-free survival is given in Table 2. Lower RT-PCR Δ*C*_t_ values were associated with strong IHC and FISH positivity. A lower RT-PCR Δ*C*_t_ was also associated with a response to crizotinib and longer progression-free survival (*p* = 0.011, HR = 1.351 (95% CI: 1.071–1.703)) (Figure 2).

One of the RT-PCR-positive wild-type *ALK* specimens (case 34, Table 2) had a Δ*C*_t_ of 4.47 and was IHC and FISH-negative. The patient presented with brain metastases and a small primary in her right lung without extracranial metastases. The patient was given whole brain radiation followed by crizotinib. The crizotinib dose was reduced because of diarrhea but nevertheless the patient had stable disease in the brain and chest without new metastases for 28 months before a mild progression in the brain and primary lesion in the lung. She was placed on ceritinib but tolerated the medication poorly due to diarrhea. The patient was then started on alectinib and remains on the drug at reduced doses with stable disease. Of note, both the patient’s brain metastasis and a biopsy of her primary lung tumor were RT-PCR-positive for *ALK*. Importantly, the lung tumor biopsy was negative for any crizotinib-responsive genetic alterations of either ROS1 or MET, including the MET exon 14 skipping mutation. This patient was the sole case of the four individuals with full-length *ALK*-expressing tumors in our series who was treated with ALK inhibitors. The scientific basis for the response of her tumors to inhibitor therapy remains unclear.

Case 32 (Table 2) was IHC 1+ but FISH-negative for a break-apart pattern. RT-PCR of this case was positive and sequencing detected an *EML4-ALK* variant 3a rearrangement. The other discordant cases that were RT-PCR and sequence-positive but IHC and FISH-negative in the study died before sequencing data was available to the clinician to determine their suitability for ALK inhibitor therapy.

### 2.4. Samples with Insufficient Tumor Content for IHC and FISH

Two patients who were consented for the study had tumor contents in their biopsy samples below the cut-off for IHC or FISH testing at the BC Cancer Agency (data not shown) and were therefore not included in the correlation analysis. However, their specimen tumor content was sufficient to be tested by RT-PCR and both were positive.

The first case was a 51-year-old female never-smoker who presented with stage IV NSCLC. Sequencing revealed an *EML4-ALK* variant 3 rearrangement. The patient (whose case has also been reported elsewhere) responded to crizotinib for 9 months before progression in the liver, requiring a change in therapy to ceritinib and stereotactic ablative radiotherapy [17]. She died from progressive disease 23 months from starting an ALK inhibitor. The second patient is an 80-year-old female smoker who presented with low volume stage IV adenocarcinoma of the lung and was started on crizotinib in 2014. She has maintained stable disease for 3 years.

## 3. Discussion

Both FISH and IHC are widely used in clinical laboratories for the detection of *ALK* rearrangements in NSCLC tumor samples. While many laboratories still use FISH as a stand-alone test, the use of IHC as an initial screening test is becoming more common. Additionally, there is good evidence to support a strongly positive IHC result (3+/3) as indicative of the presence of an *ALK* rearrangement, while confirmation of equivocal IHC results is usually reflexed to FISH [18]. Studies that have used NGS to confirm discordant IHC and FISH results suggest that FISH has a significant false-negative rate, which could deny patients access to ALK inhibitor therapies [19,20,21].

NGS fusion panels as the sole platform for detecting ALK and other NSCLC biomarkers in a single test are attractive. However, there are a wide variety of capabilities among publicly-funded and private diagnostic laboratories world-wide and many do not have access to NGS at the current time. As such, there continues to be a role for single-gene assays like RT-PCR for ALK detection in NSCLC.

RT-PCR is a sensitive and rapid detection assay when compared to FISH for the detection of *ALK* in NSCLC tumor samples. Earlier concerns of false-negative results due to allele-specific primer selection that permits detection of only a subset of the many *ALK* rearrangements have been obviated by the use of assays employing a primer set that detects the 3′ kinase domain conserved among all known *ALK* rearrangements regardless of the fusion gene partner [14,15,16]. Issues regarding fixation, storage and age of FFPE samples and their impact on RNA-based RT-PCR are also relevant for DNA and protein-based ALK detection methods. The quality of RNA in archival clinical FFPE samples has been addressed in a number of studies and shown to be suitable for molecular testing [22,23]. Of note, several samples in this report were archival and as old as 8 years but still yielded RNA of sufficient quality for detection of *ALK* expression. RT-PCR also has the advantage of a much smaller tumor content requirement than FISH [15]. Consistent with a requirement for minimal tumor tissue, most of the samples tested in the current study were endobronchial biopsies or cytology FFPE specimens rather than surgical resections.

In this study, we investigated the ability of a RT-PCR assay using a Δ*C*_t_ ≤ 8 to detect *ALK* expression in lung cancer FFPE samples from an enriched cohort of 95 patients and compared the results to FISH and IHC. RT-PCR detected all 21 cases with *ALK* rearrangements determined by FISH. In addition, eleven FISH-negative and RT-PCR-positive discordant cases were shown by sequencing to contain *ALK* rearrangements. These results are similar to previous studies, which also demonstrated a high sensitivity for the detection of *ALK* expression in NSCLC using RT-PCR-based approaches [14,15,16]. The percentage of patients in our study who were ALK-positive by RT-PCR (38%) and by RT-PCR, IHC and FISH (22%) was significantly higher than the estimated 2% to 7% reported in the literature. This was due to enrichment of our cohort of patients by excluding EGFR mutation-positive cases and using patients in the later part of the study who were known to be IHC and FISH-positive prior to enrollment.

In our study, the percentage of patients who were nonsmokers in the ALK-positive by RT-PCR, IHC and FISH group was 76% compared to 50% in the RT-PCR-positive group. Even if we exclude the 4 wild-type cases in the RT-PCR group who were all 4 smokers, there remains a significant difference. Our cohort of patients in the RT-PCR-positive group was small and included cases already known to harbor ALK rearrangements by IHC and FISH, so this may have resulted in some selection bias. A larger study is needed to determine if there is a real discrepancy in rates of smoking between the RT-PCR-positive and RT-PCR/IHC/FISH-positive patients.

A recently reported study of the same ALK RGQ RT-PCR Kit used in our study utilized a Δ*C*_t_ ≤ 3.5 and employed FISH as a reference for determining the accuracy of the test [16]. The RT-PCR assay was shown in their cohort of mostly lung cancer resection samples to have a sensitivity and specificity of 100% [16]. However, when we applied a Δ*C*_t_ ≤ 3.5 cut-off to our series of samples we found 12 false-negative cases that were shown by FISH or sequencing to harbor *ALK* rearrangements. Only two of these 12 cases were treated with crizotinib but both demonstrated responses. The differences in Δ*C*_t_ assay cut-off values can be attributed to the analytical reference tests used to establish true status. The Δ*C*_t_ ≤ 8 cut-off was determined in order to minimize false-positive and negative results relative to *ALK* expression as determined by sequencing on archival specimens, whereas the Δ*C*_t_ ≤ 3.5 cut-off was established using *ALK* FISH as a reference.

Four of the 36 RT-PCR-positive samples in our study were shown be due to transcriptional upregulation of *ALK*. All 4 cases were IHC and FISH-negative. One of these patients was treated with crizotinib and had stable disease for 28 months followed by mild progression that responded to the second-generation ALK inhibitor alectinib. The patient’s tumor was tested for the alternative drivers ROS1 and MET that are known to be responsive to some ALK inhibitors but the results were negative. Gruber et al. [14] also found full-length *ALK* transcript expression in 1.1% of NSCLC tumors but none of their patients were treated with ALK inhibitors; of note, similar to our cases, the full-length ALK-positive tumors reported by Gruber and co-workers expressed little or no ALK protein (0 or 1+) on IHC. Since RT-PCR is measuring mRNA, it is possible that either the transcripts are unstable or translation is inefficient, thus resulting in the lack of detection of protein. It is important to note that IHC, like the RT-PCR assay used in this report, is not capable of differentiating ALK fusion-positive versus full-length ALK-positive NSCLC; thus, occasional cases of IHC-positive lung cancer could possibly express full-length ALK rather than the chimeric kinase although none of our 4 cases were IHC-positive. RT-PCR assays that assess the balance between expression of 5′ *ALK* (representing transcripts indicative of the full-length receptor tyrosine kinase) relative to 3′ *ALK* transcripts (that are common to both full-length and fusion forms of *ALK*) have been designed for this discrimination [14,24]; however, the ALK RGQ RT-PCR Kit targets only the 3′ region of the *ALK* transcript which encodes the kinase domain. Amplification or increased copy number of full-length *ALK* has been reported in NSCLC tumors [25], but is not thought to be an oncogenic driver. In our cohort of patients we detected an increased in *ALK* gene copy number and amplification in both RT-PCR-positive and negative samples but none in the 4 cases with transcriptional upregulation of full-length *ALK*. Activating point mutations of full-length *ALK* occur in the pediatric malignancy neuroblastoma and in certain thyroid carcinomas [26,27,28]; however, sequencing of full-length *ALK* in our NSCLC cases revealed no evidence for oncogenic point mutations. Thus, it remains unclear why our patient has had a prolonged response to ALK inhibitors.

Unlike recently reported studies evaluating the performance of RT-PCR [14,15,16], several tumor samples used in our study were obtained from extrathoracic NSCLC metastatic lesions, including 7 samples from brain. Three of the four cases with full-length *ALK* transcripts were from brain metastases. *ALK* gene amplification was not noted on FISH for any of these samples. The significance of this finding is not clear, since another RT-PCR-positive brain sample (case 33) was shown to contain a *KIF5B-ALK* fusion, while three RT-PCR-negative samples were also from brain metastases. The frequency of *ALK* fusions in brain metastases is similar to what is seen in primary tumors from the lung; however, amplification of the full-length *ALK* gene appears to be increased in brain metastases without ALK fusions compared to the primary tumor in the same patients [29]. Of note, *ALK* amplification is often not associated with ALK protein overexpression [29]; in such situations, of course, ALK would not be expected to be a driver of the malignancy. It is also important to note that brain is one of few sites in the body where wild-type ALK is normally expressed [30]. Thus, in cases where metastatic samples from brain are tested as the initial screen for *ALK* fusions and found to be RT-PCR-positive, it may be useful to also test a sample from the lung primary tumor or to reflex the metastatic sample to NGS.

Although the correlation of RT-PCR test results with patient outcomes after treatment of positive cases with ALK inhibitors was done retrospectively, we were able to obtain inhibitor response data on all patients determined to be FISH-positive and to compare this with RT-PCR and IHC results. Our cohort of 21 patients was small, but the data suggest that RT-PCR-positive cases with the lowest Δ*C*_t_ values that were also FISH and IHC 3+ positive were more likely to have a prolonged progression-free survival (Figure 2). Response data obtained retrospectively and outside a clinical trial setting must be interpreted with caution as it is influenced not only by the presence of an *ALK* rearrangement but potentially also by multiple additional variables including the extent of disease and performance status of the patients at the start of ALK inhibitor treatment, brain metastases, and the toxicity from the drug. In a prior study comparing IHC, FISH, and RT-PCR for ALK detection in NSCLC the authors found that samples with a high abundance of *ALK* transcript expression were also IHC strongly positive and FISH-positive [31]. Unfortunately, no ALK inhibitor response was provided as part of that study.

In our patient cohort, we found 9 RT-PCR-positive cases that were IHC and FISH-negative, as well as 2 RT-PCR-positive, IHC 1+/FISH-negative cases. Our study was not designed to prospectively treat patients with RT-PCR-positive/IHC or FISH-negative cases with an ALK inhibitor, so there was only one patient (case 32, Table 2) treated of the 11 FISH-negative discordant cases who was RT-PCR-positive with an identified *ALK* rearrangement by sequencing. This patient’s tumor contained an *EML4-ALK* variant 3a fusion, which has been reported to be relatively resistant to targeted therapy compared to other ALK fusion variants [32], and failed to respond to both crizotinib and the second-generation ALK inhibitor, ceritinib. Although there have been a small number of cases reported in the literature that had *ALK* rearrangements detected by RT-PCR but not FISH who responded to treatment with an ALK inhibitor [33], the functional significance of RT-PCR-positive tumors with proven genomic rearrangements that are FISH and/or IHC-negative will need to be demonstrated in large prospective trials in which an ALK inhibitor is given to both cohorts. Matsson et al. [9] reported 3 cases out of 6 that were D5F3 IHC-negative and FISH-negative but ALK gene expression-positive. Their study did not report any treatment response data and the authors did not speculate on why they detected ALK gene expression but without detection of a rearrangement by FISH or ALK protein by IHC. They suggest that there may be tissue heterogeneity between whole resection specimens versus smaller biopsies and that might influence IHC results. Nine of the 11 discordant cases in our study were biopsies.

NGS-positive/IHC-negative/FISH-negative cases appear to be less common compared to NGS-positive/IHC-positive/FISH-negative results [20]. Ali et al. [21], reported a NGS-positive, IHC and FISH-negative case that had a durable response to an ALK inhibitor (28 months). IHC was negative using both the D5F3 and 5A4 ALK antibodies. The authors were unable to offer an explanation for the prolonged response to an ALK inhibitor when IHC was unable to detect the ALK protein.

Of note, the discordant cases in our study had higher Δ*C*_t_ values (mean = 4.98 vs. 0.84 for the concordant cases) which is indicative of lower levels of *ALK* RNA expression and may result in lower levels of ALK protein expression. Our response data (Figure 2) suggest that these cases maybe somewhat less likely to respond well to an ALK inhibitor but further studies are needed. The *ALK* fusions detected by sequencing in our study have been reported to be positive by IHC or FISH by other investigators, so this is also not a likely explanation for our discordant cases.

The 100% sensitivity of the ALK RT-PCR Kit for the identification of ALK-positive NSCLC cases, as shown here and by others independently [15,16], supports the use of the test as a screening approach for ALK in newly diagnosed lung cancer. The ability to format the assay for high-throughput analysis (68 samples per run) with rapid turnaround (within 4 h of sectioning), together with automated interpretation of results, also argue favorably for use of the test when compared to FISH and IHC for ALK screening in NSCLC. The specificity of the ALK RGQ RT-PCR KIT in this study compared to FISH was also been shown to be high (94%) in cases lacking full-length *ALK* expression, and 100% in two other independent studies [15,16]. Based on the cumulative experience with the ALK RT-PCR Kit, we propose two possible diagnostic algorithms. RT-PCR could be used as the front-line screen for ALK in newly diagnosed NSCLC. Cases that score negative would not be eligible for ALK inhibitor therapy. Those cases that were positive could be reflexed for corroboration to IHC to detect ALK protein expression or NGS for the identification of specific *ALK* fusions. Alternatively, the RT-PCR assay is also ideal for reflex confirmation of tumor samples that are initially screened by IHC or FISH but with an equivocal result. Marchetti et al. [16] has also suggested that tumor samples that have been screened by IHC and are negative but with clinicopathological characteristics associated with ALK fusions (nonsmokers, signet ring cell carcinoma, TTF1-positive) could be reflex tested by RT-PCR.

## 4. Materials and Methods

### 4.1. Patients and Tumor Samples

The studies reported in this article were performed from December 2013 to August 2015 during the PCRTALK (PCR tumor ALK) trial (Clinicaltrials.gov ID: NCT02010047). Formalin-fixed paraffin-embedded (FFPE) biopsy, cytology and resection samples from an enriched cohort of patients with advanced non-squamous cell, non-neuroendocrine lung tumors were screened for ALK fusions using IHC. The study cohort was enriched by selecting only patients whose tumor samples were EGFR wild-type. We also used some samples that were known to be ALK-positive by IHC and FISH prior to consenting patients to the study to ensure we had an adequate number of ALK-positive samples for comparative analyses between the three modalities of ALK testing. Only patients with stage IIIB or IV NSCLC were included in the study. Tumors with a positive IHC test were reflexed to FISH. ALK-positive and -negative samples were then tested by RT-PCR. This study was conducted in accordance with the Declaration of Helsinki and the protocol was approved by the British Columbia Cancer Agency Research and Ethics Board (H13-01763). Patients participating in the study were required to sign an informed consent. Consent for testing of archival specimens from deceased patients was deemed unnecessary by the Ethics Board. Those patients with tumors that harbored an ALK fusion as determined by a strong positive IHC result and reflex FISH confirmation were eligible for treatment with the first-generation ALK inhibitor crizotinib.

NSCLC tumor samples with less than 10% tumor content and cellularity were rejected by the BC Cancer Agency Molecular Laboratory for further testing. If patients did not have additional FFPE blocks with sufficient tumor content for testing, in some cases a new biopsy was requested.

### 4.2. Immunohistochemistry

Immunohistochemistry was performed on 4 μm sections, using the Novocastra mouse monoclonal antibody p80 ALK (Clone 5A4, NCL-ALK, Leica, Wetzlar, Germany) at 1:25 dilution for 52 min. Staining was performed using the UltraView DAB universal detection kit (Roche, Basel, Switzerland) including an Amplification Kit (Roche), and was performed on a Benchmark ULTRA autostainer (Roche). Positive controls included lung tumor confirmed by FISH to be positive for *ALK* rearrangement. Negative controls included lung tumor confirmed by FISH to be negative for rearrangement as well as non-tumor lung tissue. Expression of all ALK antibodies on each tissue section was assessed and scored by an experienced pulmonary pathologist. The pathologist scored each tumor slide qualitatively by determining the intensity of cytoplasmic staining, as follows: 0, no staining; 1+, weak/faint cytoplasmic staining; 2+, moderate cytoplasmic staining; and, 3+, intense cytoplasmic staining. Cases with 1+, 2+ and 3+ staining were regarded as “positive” and reflexed to FISH for confirmation. Cases with no staining were regarded as negative.

### 4.3. Fluorescence in Situ Hybridization

FISH analysis was performed on 100 nuclei from paraffin embedded tissue sections using a Vysis ALK Break Apart FISH Probe Kit (Abbott Molecular, Des Plaines, IL, USA) according to the manufacturer’s instructions. A sample was considered positive for an *ALK* rearrangement when 15% or greater of nuclei had a separation of the orange and green signals by two signal diameters. Alternatively, a single orange (deletion of green signal) in addition to fused or broken apart signals were also indicative of the presence of an *ALK* rearrangement.

### 4.4. ALK RT-PCR

The ALK RGQ RT-PCR Kit (QIAGEN, Manchester, UK) is a one-step reverse transcription real-time polymerase chain reaction assay that detects the expression of mRNA encoding the *ALK* tyrosine kinase domain after qualification by an endogenous control (ABL) reaction (Figure 1). The ∆*C*_t_ cut-off of ≤8 was established by the manufacturer. Details regarding the method for determining the cut-off of the RT-PCR kit can be found in QIAGEN’s ALK RGQ RT-PCR Kit Handbook [34]. The kit comprises two separate assays in one tube (duplex reaction): one ALK kinase-specific assay (Green channel—FAM) and one ABL control assay (Yellow channel—HEX). The input for the kit is total RNA extracted from non-small cell lung cancer (NSCLC) FFPE tumor specimens using the RNeasy FFPE Extraction Kit (QIAGEN). Testing was performed using a RotorGene Q 5-plex +HRM system (QIAGEN). A neat input of 5 microlitres of total RNA from each specimen was used in the assay. Since the quantity of RNA is not determined prior to RT-PCR, the *C*_t_ value in the Yellow channel for the ABL assay is used to determine the quality and quantity of input RNA. Based on the specifications outlined in the kit handbook, if the *C*_t_ value for the ABL assay falls within an acceptable range, and the ∆*C*_t_ (ALK reaction *C*_t_ value in the Green channel—ABL reaction *C*_t_ value in the Yellow channel) is ≤ 8, then the sample is ALK expression-positive. A positive control containing in vitro transcript (IVT) mix of both ALK and ABL target sequence is included in the ALK Kit to verify assay performance.

### 4.5. Sanger Sequencing

Extracted RNA (RNeasy FFPE Kit, QIAGEN) from the patient biopsy was reverse transcribed to cDNA using a reverse primer sequence of 5′-TTGCTCAGCTTGTACTCAGGGCT-3′ and used in a 5′ Rapid Amplification of cDNA Ends (RACE) PCR followed by Sanger sequencing to identify the ALK fusion partner. Full length *ALK* expression was identified by the presence of *ALK* coding sequence 5′ to the highly-conserved break-point of *ALK* at the junction of exons 19–20 at position +3172 in the transcript. If *ALK* sequence was present juxtaposed to the break-point, the sample was designated wild-type. Sequencing of cDNA encoding the ALK kinase domain was performed by targeting exons 23–25 of *ALK* encoding a portion of kinase domain by PCR with M13-tagged primers. The resulting amplicon was subjected to Sanger sequencing.

### 4.6. Next-Generation Sequencing

At the initiation of the study, the Sanger sequencing method described above was used to determine ALK fusion status, but a targeted RNA panel for next-generation sequencing became available shortly thereafter. A subset of samples with sufficient cDNA remaining was tested by both strategies to bridge methods. Extracted RNA (50 ng) from the patient biopsy was reverse transcribed to cDNA using a mixture of random hexamers and poly-dT oligonucleotides. The cDNA was then hybridized to a custom oligonucleotide pool containing triplicate upstream and downstream oligonucleotides specific for *ALK* (9 positions across transcript) and 43 unique ALK fusion events (TruSeq Targeted RNA Expression Panel, Illumina, San Diego, CA, USA). The hybridized oligo-nucleotides were then bound to paramagnetic streptavidin beads. The unbound oligonucleotides were washed away and remaining hybridization products were subjected to an extension-ligation. The extension-ligation products were amplified using primers that added index sequences for sample multiplexing as well as common adapters. PCR products were purified and pooled before sequencing on the MiSeq platform (Illumina).

### 4.7. Statistical Analysis

Computations were performed using R version 3.2.3 (R Foundation for Statistical Computing, Vienna, Austria). Confidence intervals (CIs) for sensitivity and specificity were computed using the R function ‘binom.test’. The R ‘survival’ package was used to fit a Cox proportional hazards model to progression-free survival to determine its association with Δ*C*_t_ [35]. The result was expressed as a hazard ratio (HR) with 95% confidence interval.

## 5. Conclusions

The results of our study provide further confirmation of RT-PCR as an efficient and reliable diagnostic screening approach for the detection of ALK in NSCLC samples.

## Figures and Tables

**Table 1:** Clinical and pathological characteristics of cases tested by ALK RT-PCR in 95 patients.

ALK RT-PCR			ALK RT-PCR, IHC and FISH
Clinicopathological Characteristics	Positive	Negative	Positive
Total Number of Cases	36	59	21
Median age	69 (36–81)	69.5 (43–82)	67 (36–81)
Gender			
Male	14 (39%)	26 (44%)	6 (29%)
Female	22	33	15
Sample site			
Lung primary	19 (52%)	36 (61%)	10 (48%)
Metastatic	17 (47%)	23 (39%)	11 (52%)
Sample type			
Resection	9 (25%)	14 (24%)	3 (14%)
Lung	4	11	2
Brain	4	3	
Kidney	1		1
Biopsy and cytology	27 (75%)	45 (76%)	18 (86%)
Histology			
Adenocarcinoma	34 (95%)	59 (100%)	19 (90%)
Adenosquamous	2	0	2
Stage			
I-IIIA	0	0	0
IIIB-IV	36	59	21
Smoking history			
Never or light smoker	18 (50%)	1 (1.7%)	16 (76%)
Smoker	18	58	5
EGFR mutations			
Wild-type	36	59	21
Mutant	0	0	0

**Table 2:** Correlation of ALK immunohistochemistry (IHC) and fluorescence in situ hybridization (FISH) with RT-PCR-positive cases (Δ*C*_t_ ≤ 8).

Case	Sample	Tumor Content (%)	IHC (0,1,2,3)	FISH (% Positive)	RT-PCR Δ*C*_t_	ALK Fusion by Sequencing	Response to First-Line ALK Inhibitor and PFS (Months)
1	B	60	3	+(66)	−65	EML4-Var1 (2)	+(6)
2	B	95	2	+(78)	−0.07	EML4-Var1 (1,2)	+(30)
3	B	70	3	+(59)	0.09	EML4-Var1 (1)	+(22)
4	R	90	2	+(21)	0.22	EML4-Var1 (2)	+(20)
5	B	90	3	+(96)	0.58	EML4-Var1 (1,2)	+(4)
6	C	40	3	+(31)	0.06	EML4-Var1 (2)	+(24)
7	R	95	3	+(66)	0.41	EML4-Var3a (2)	+(23)
8	B	>50	3	+(53)	1.09	EML4-Var3a (2)	+(9)
9	B	90	3	+(52)	2.11	EML4-Var3a/5 (2)	No Response
10	B	50	3	+(16)	1.29	EML4-Var3b (2)	+(7)
11	R	90	3	+(65)	−0.83	ND	+(24)
12	B	80	3	+(94)	2.69	ND	+(19)
13	C	>50	3	+(34)	1.19	QNS	+(4)
14	B	50	3	failed twice	0.21	QNS	+(20)
15	B	85	1	+(38)	7.12	QNS	+(6)
16	R	80	3	+(84)	−0.97	QNS	+(30)
17	C	60	3	+(73)	−0.64	QNS	+(33)
18	B	60	2	+(58)	−0.51	QNS	+(3)
19	B	80	2	+(56)	−0.11	QNS	+(12)
20	B	50	3	+(40)	3.62	QNS	No Response
21	B	70	ND	+(25)	0.80	EML4-Var1 (1,2)	+(48)
22	R	80	1	−(0)	5.63	EML4-Var1 (2)	NT
23	B	70	0	−(6)	3.45	EML4-Var1 (1,2)	NT
24	B	50	0	−(2)	4.35	EML4-Var1 (1,2)	NT
25	B	90	0	−(0)	5.22	EML4-Var1 (1,2)	NT
26	B	50	0	−(0)	5.22	EML4-Var1 (1)	NT
27	B	95	0	NT	4.57	EML4-Var2 (2)	NT
28	B	80	0	−(6)	6.83	EML4-Var3a (1)	NT
29	R (brain)	90	0	−(6)	4.83	KIF5B (2)	NT
30	B	50	0	−(13)	5.81	KIF5B (2)	NT
31	R	60	0	−(0)	4.07	KIF5B (2)	NT
32	C	60	1	−(8)	4.79	EML4-Var3a (1)	No Response
33	R (brain)	90	0	−(0)	3.98	Wild-Type (1,2)	NT
34	B (brain)	90	0	−(0)	4.47	Wild-Type (1,2)	+(28)
35	B	90	0	−(2)	5.1	Wild-Type (1)	NT
36	B (brain)	95	0	−(0)	5.22	Wild-Type (2)	NT

B, biopsy; R, resection; C, cytology; QNS, quantity not sufficient; ND, not done; PFS, progression-free survival; NT, not treated with an ALK inhibitor. ALK fusion confirmed by RACE with Sanger sequencing (1), targeted RNA next generation sequencing (2), or both (1,2).

**Table 3:** Sensitivity and specificity of RT-PCR using Δ*C*_t_ ≤ 8 and Δ*C*_t_ ≤ 3.5 with FISH as the reference standard for detecting *ALK* rearrangements.

**RT-PCR**	**FISH+**	**FISH−**	**Total**
**Δ*C*_t_ cut-off of ≤8**			
**Positive**	21	15	36
**Negative**	0	59	59
**Total**	21	74	95
Sensitivity = 100% (95% CI: 84–100%)			
Specificity = 80% (95% CI: 69–88%)			
**RT-PCR**	**FISH+**	**FISH−**	**Total**
**Δ*C*_t_ cut-off of ≤3.5**			
**Positive**	19	1	20
**Negative**	2	73	75
**Total**	21	74	95
Sensitivity = 90% (95% CI: 70–99%)			
Specificity = 99% (95% CI: 93–100%)			

**Table 4:** Sensitivity and specificity of RT-PCR using Δ*C*_t_ ≤ 8 and Δ*C*_t_ ≤ 3.5 with FISH plus sequencing as the reference standard for detecting *ALK* rearrangements.

**RT-PCR**	**FISH or Sequencing+**	**FISH− or Sequencing−**	**Total**
**Δ*C*_t_ cut-off of ≤8**			
**Positive**	32	4	36
**Negative**	0	59	59
**Total**	32	63	95
Sensitivity = 100% (95% CI: 89–100%)			
Specificity = 94% (95% CI: 85–98%)			
**RT-PCR**	**FISH+ or Sequencing+**	**FISH− or Sequencing−**	**Total**
**Δ*C*_t_ cut-off of ≤3.5**			
**Positive**	20	0	20
**Negative**	12	63	75
**Total**	32	63	95
Sensitivity = 62% (95% CI: 44–79%)			
Specificity = 100% (95% CI: 94–100%)			
